# Supplementary material for: Complexome profiling of the Chlamydomonas psb28 mutant reveals TEF5 as an early PSII assembly factor
Source: Plant Cell. 2025 Mar 17;37(6):koaf055. doi: 10.1093/plcell/koaf055 (PMC12164590; doi:10.1093/plcell/koaf055)
Supplement: koaf055_Supplementary_Data [file koaf055_supplementary_data.zip › psb28_interactive.zip!/plots/intensity/qualityPlots/peptides/SNR1_Cre01.g000250.t1.2.html]

### Cre01.g000250.t1.2

Trivial name: SNR1  
SeqCoverage [median]: 7.90  
Mass: 24.83 kDa  
  
 

| Sequence | MissedCleavage | Mass [Da] | wtIntensity | muIntensity | Score |
| --- | --- | --- | --- | --- | --- |
| AINAAASK | 0 | 744.41 | 2281.20 | 3089.71 | 114.50 |
| ALEEQEQEQQGK | 0 | 1415.65 | 1118.94 | 2362.39 | 240.82 |
|  |  |  | 3400.1 | 5452.1 |  |
